# Quantity and Volume of Perivascular Spaces Are Inversely Associated With Multiple Sclerosis Relative to Cerebrovascular Disease and Migraine

**DOI:** 10.1002/acn3.70193

**Published:** 2025-09-15

**Authors:** Elle M. Levit, Elizabeth A. Horwath, Daniel L. Schwartz, Lisa C. Silbert, Russell T. Shinohara, Andrew J. Solomon

**Affiliations:** ^1^ Department of Neurological Sciences University of Vermont Burlington Vermont USA; ^2^ Department of Biostatistics, Epidemiology, and Informatics, Penn Statistics in Imaging and Visualization Center University of Pennsylvania Perelman School of Medicine Philadelphia Pennsylvania USA; ^3^ Department of Neurology, Oregon Alzheimer's Disease Research Center Oregon Health & Science University Portland Oregon USA; ^4^ Advanced Imaging Research Center Oregon Health & Science University Portland Oregon USA

**Keywords:** multiple sclerosis, neuroimaging, neuroinflammation, perivascular spaces

## Abstract

**Objective:**

To quantify the number and volume of whole brain perivascular spaces (PVS) using a detection and segmentation algorithm in participants with multiple sclerosis (MS) and patients with disorders mimicking MS known to potentially influence PVS, such as cerebrovascular disease.

**Methods:**

T1‐weighted and T2‐weighted FLAIR sequences were obtained on 3T MRI from 40 participants: 10 with MS, 10 with MS and a known comorbidity associated with MRI white matter abnormalities, 10 with migraine and MRI T2 hyperintense lesions, and 10 who were previously misdiagnosed with MS due to a variety of diagnoses. MRIs were analyzed using a previously validated automated segmentation pipeline. Primary outcomes included PVS number and volume, which were evaluated separately in each model.

**Results:**

MS diagnosis was inversely associated with the number of PVS (*t*(23.99) = 3.92, *p* < 0.001) and PVS volume (*t*(22.49) = 3.64, *p* < 0.001). ROC analysis demonstrated an AUC above 0.8 for both the number of PVS and PVS volume for differentiating the MS and non‐MS cohorts. In logistic regression, the number of PVS (OR = 0.98, 95% CI [−0.03, −0.01], *p* < 0.05) and volume of PVS (OR = 0.98, 95% CI = [0.97, 0.99], *p* < 0.006) were significantly inversely associated with MS diagnosis.

**Interpretation:**

These findings suggest that those with a confirmed diagnosis of MS had a lower PVS burden compared to individuals with migraine or misdiagnosis of MS, irrespective of vascular comorbidities. The degree to which cerebrovascular disease influences PVS in patients with MS and other diagnoses warrants further study in larger longitudinal cohorts.

## 1 Introduction

Multiple sclerosis (MS) is characterized by central nervous system (CNS) inflammation leading to demyelination and axonal damage with subsequent neuronal loss [1, 2]. The immunological features and location of CNS neuroinflammation in MS are variable and influenced by disease stage. Blood–brain barrier (BBB) disruption and lymphocyte infiltration are predominant in early disease while CNS‐specific innate immune cell response prevails in later progressive disease, although some degree of both types of neuroinflammation may be present at any stage of disease [3].

Prior work in MS has demonstrated that inflammatory cells survive in the CNS and become trapped in perivascular spaces (PVS), the leptomeninges, and potentially the choroid plexus [3, 4, 5]. Sequestered inflammation in MS has been associated with clinical outcomes and appears to persist despite high efficacy disease‐modifying therapy (DMT) use, and therefore has become a growing area of interest for the detection and quantification of MS disease activity [6, 7]. PVS are involved in neuroimmune functions and clearance of metabolites via the glymphatic system, and enlargement of these spaces may indicate dysfunction of normal processes and contribute to disease pathology [8, 9].

In MS, PVS can become enlarged and are detectable on MRI during active inflammation, correlating with MRI contrast‐enhancing lesions that are indicative of BBB disruption [10, 11]. Some MRI studies have shown that enlarged PVS are more prevalent, higher in number, and larger in volume in MS patients compared to healthy controls [10, 12]. While age is an independent risk factor for enlarged PVS [13], it has been shown that PVS in MS may be enlarged independent of age or age‐related brain volume changes [10]. However, enlargement of PVS has been associated with inflammatory and noninflammatory conditions such as hypertension, small vessel cerebrovascular disease, and systemic rheumatologic disease such as SLE [9, 14]. Some data also suggest that enlarged PVS in MS may be more reflective of microangiopathic changes [13]. A recent 7T MRI study found a nonsignificantly higher number of PVS in subjects without MS compared to MS, raising further uncertainties of the relationship between PVS and MS [15]. Though there is a paucity of data evaluating an association between PVS and clinical outcomes in MS, one study found higher frequency of PVS in the basal ganglia was associated with an increased risk of disability [8]. Manual quantification of PVS is exceptionally time consuming, and more recently, time‐efficient methods employing automated computational quantification of PVS have been developed and validated [16, 17].

The aim of this exploratory pilot study was to use PVS quantification to compare the frequency and size of PVS in patients with MS to patients without MS, while including participants in each cohort with and without comorbidities known to influence PVS.

## 2 Methods

### 2.1 Participant Selection

Forty participants in a cross‐sectional study where patients received their routine care comprising four cohorts participated in the study [18]. These included (1) 10 with a diagnosis of MS by 2017 criteria [19] and no history of a comorbidity that may also cause MRI white matter abnormalities (“MS”); (2) 10 with a diagnosis of MS by 2017 criteria and at least one additional comorbidity known to cause MRI white matter abnormalities (“MS+”); (3) 10 with a diagnosis of migraine and a previous history of an MRI with at least two white matter abnormalities in any location and without additional known vascular risk factors or risk factors for MRI abnormalities (“migraine”); and (4) 10 who had been previously incorrectly diagnosed with MS, who did not fulfill 2017 diagnostic criteria for MS, and in whom a variety of diagnoses had been identified to explain clinical and radiographic abnormalities mistaken for MS (“misdiagnosed”), including migraine, small vessel ischemic disease (SVID), and vitamin B12 deficiency. Diagnoses of participants in the migraine cohort were established after evaluation by a neurologist. For this analysis, MS and MS+ combined defined the “total MS cohort,” and migraine and misdiagnosed combined defined the “non‐MS cohort.”

### 2.2 Imaging

T2‐weighted FLAIR (1‐mm isotropic resolution) was acquired on a 3T Philips dStream MRI with 32‐channel head coil as well as T1‐weighted sequence (0.8‐mm isotropic resolution) with 3D sagittal acquisition of the entire brain.

### 2.3 Automated PVS Assessment

T1 and FLAIR‐weighted volumes in NIFTI format were used to segment MR‐visible PVS in cerebral WM. WM masks were generated by Freesurfer (recon‐all, v7.1.1) [20] after using ANTs brain extraction to strip extracerebral tissue (antsBrainExtraction.sh) [21]. T1 and FLAIR‐weighted volumes were co‐registered using flirt (FSL v6.0) [22] and were submitted to the SAMSEG lesion segmentation tool (run_samseg, v7.1.1) [23] to isolate lesions. Holes in WM masks were filled, and the resultant masks were eroded by one voxel to avoid false alarms caused by partial volume effects from neocortex, deep GM structures, and ventricles (AFNI v22.2.08, 3dmask_tool, 3dcalc) [24]; lesion masks were also removed, and the final eroded and lesion‐eliminated WM mask was used as the search space for two‐step neighborhood heterogeneity‐based MR‐visible PVS segmentation [25]. All masks and resulting segmentations were manually reviewed for gross errors.

### 2.4 Statistical Analysis

All statistical analyses were conducted using R version 4.4.2. Three sets of analyses were performed to examine the relationship between PVS and MS diagnosis. The primary outcomes of interest were the number of PVS clusters and PVS volume, which were evaluated separately in each model.

First, we conducted a receiver operating characteristic (ROC) analysis to evaluate the diagnostic accuracy of the number of PVS and volume in classifying individuals with and without MS. We report the area under the ROC curve (AUC) to quantify classification performance. Second, group differences in PVS measures were assessed using two‐sample *t*‐tests in which we compared the number of PVS and PVS volume between people with and without MS. Finally, we further evaluated the relationship between PVS measures and MS diagnosis using logistic regression. We fit univariate models to assess the independent relationship of each PVS measure with MS, followed by multivariate models controlling for confounding by age.

## 3 Results

Baseline participant demographics are presented in Table 1. Age did not differ among the four cohorts (ANOVA *p* = 0.24). 8/10 and 9/10 participants in the MS and MS+ cohorts were taking MS DMT at the time of participation. The comorbid conditions known to cause MRI white matter abnormalities in the MS+ cohort included: migraine (4), hypertension (3), hypertension and migraine (1), diabetes mellitus and migraine (1), diabetes mellitus and hypertension (1). In addition, 6/10 participants in this cohort had a comorbid history of tobacco use. The alternative diagnoses in the misdiagnosed cohort included: migraine (8), functional neurological disorder (4), trigeminal neuralgia (1), B12 deficiency (1), vertigo (1), and transient numbness (1). Diagnoses presumed responsible for abnormal brain MRI findings in this cohort included: migraine (8), small vessel ischemic disease (SVID) due to hypertension (2), SVID due to tobacco use (7), and vitamin B12 deficiency (1).

**TABLE 1:** Study participant characteristics. Values for age and years since clinical onset of MS are given as mean (standard deviation).

**MS (*n* = 10)**	
Age	44 (16)
Sex	9 F/1 M
Years since clinical onset of MS	9 (7)
Phenotype	10/10 RRMS
**MS+ (*n* = 10)**	
Age	43 (9)
Sex	9 F/1 M
Years since clinical onset of MS	9 (6)
Phenotype	10/10 RRMS
**Migraine (*n* = 10)**	
Age	47 (13)
Sex	10 F
**Misdiagnosed (*n* = 10)**	
Age	53 (7)
Sex	9 F/1 M

**FIGURE 1:**
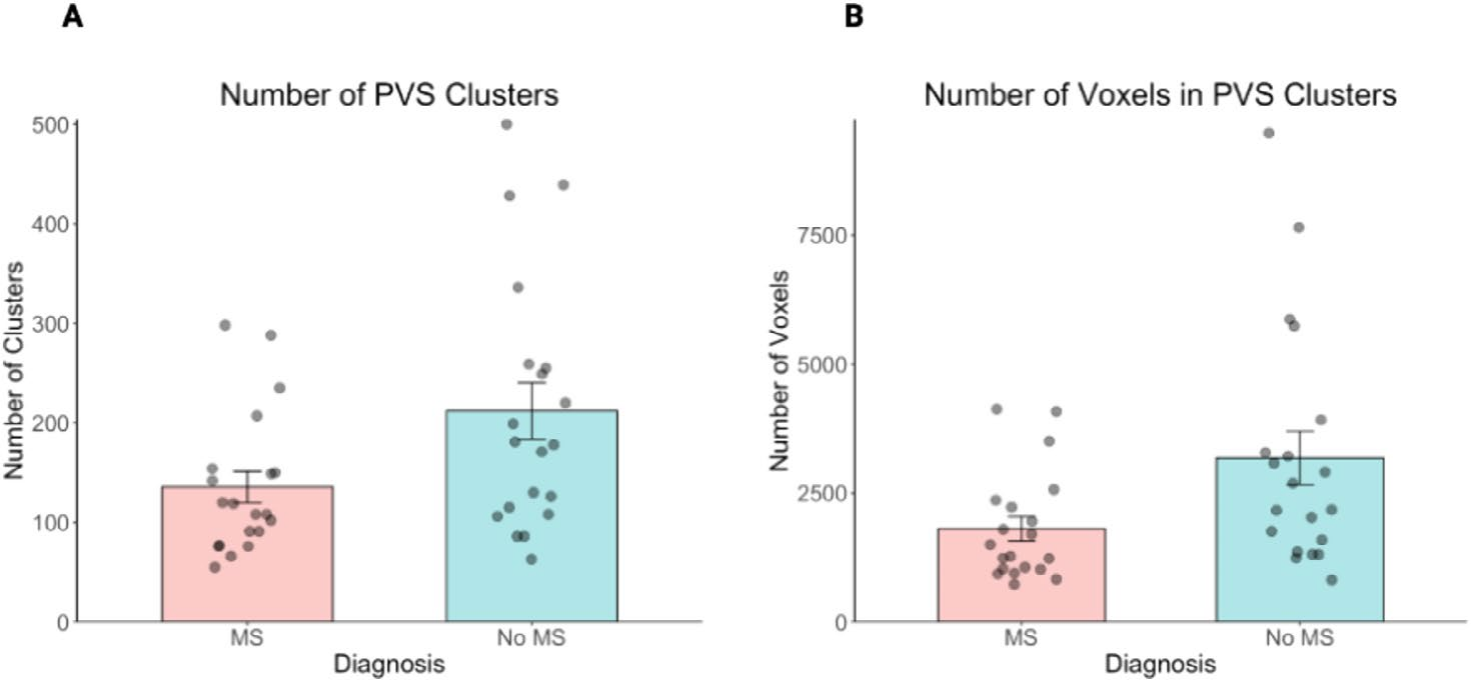
Number of PVS clusters (A) and PVS volume (B) in those with and without MS.

For each subject, the PVS segmentation pipeline identified the number of MR‐visible PVS and calculated total PVS volume. Those with a confirmed MS diagnosis had a lower burden of PVS, including fewer PVS clusters (*p* < 0.001) and fewer voxels (*p* = 0.001) (Table 2, Figure 1). The relationships between age and the number of clusters and PVS volume were not significant.

**TABLE 2:** Two‐sample *t*‐test comparing the number of clusters and voxels in total MS cohort and non‐MS cohort.

	No MS		MS		95% CI of mean difference	*t*	df	*p*
	Mean	SD	Mean	SD				
Number of PVS	231.05	123.05	116.30	45.00	54.28–175.22	3.92	23.99	0.0007
PVS volume	3.45	2.25	1.54	0.69	0.83–3.00	3.64	22.49	0.0014

**FIGURE 2:**
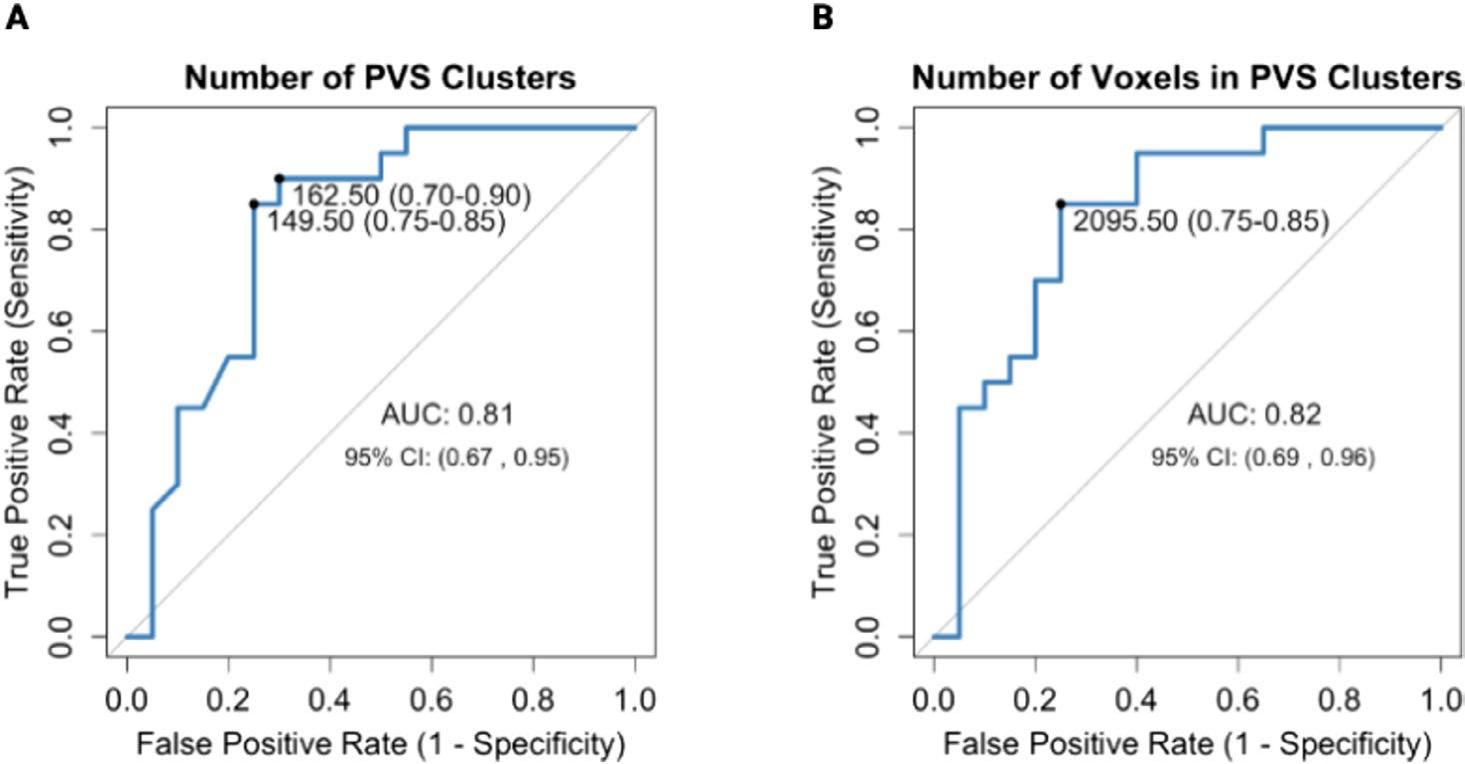
ROC analysis of the number of PVS clusters (A) and PVS volume (B) in those with and without MS.

ROC analysis revealed AUC above 0.8 for both the number of PVS clusters (CI 0.67, 0.95) and PVS volume (CI 0.69, 0.96), supporting the assertion that both the number of PVS clusters and PVS volume have high diagnostic accuracy in distinguishing people with and without MS in this cohort (Figure 2).

In our logistic regression analysis, both the number of PVS and PVS volume were significantly associated with MS diagnosis. Specifically, for each additional MR‐visible PVS cluster, the odds of having MS decreased by 0.98 (*p* = 0.005), and for each additional mL of cluster volume, the odds of having MS decreased by 0.28 (*p* = 0.006). These findings suggest that a higher burden of PVS clusters and greater PVS volume are associated with lower odds of MS diagnosis. Notably, these findings remained significant after controlling for age.

**FIGURE 3:**
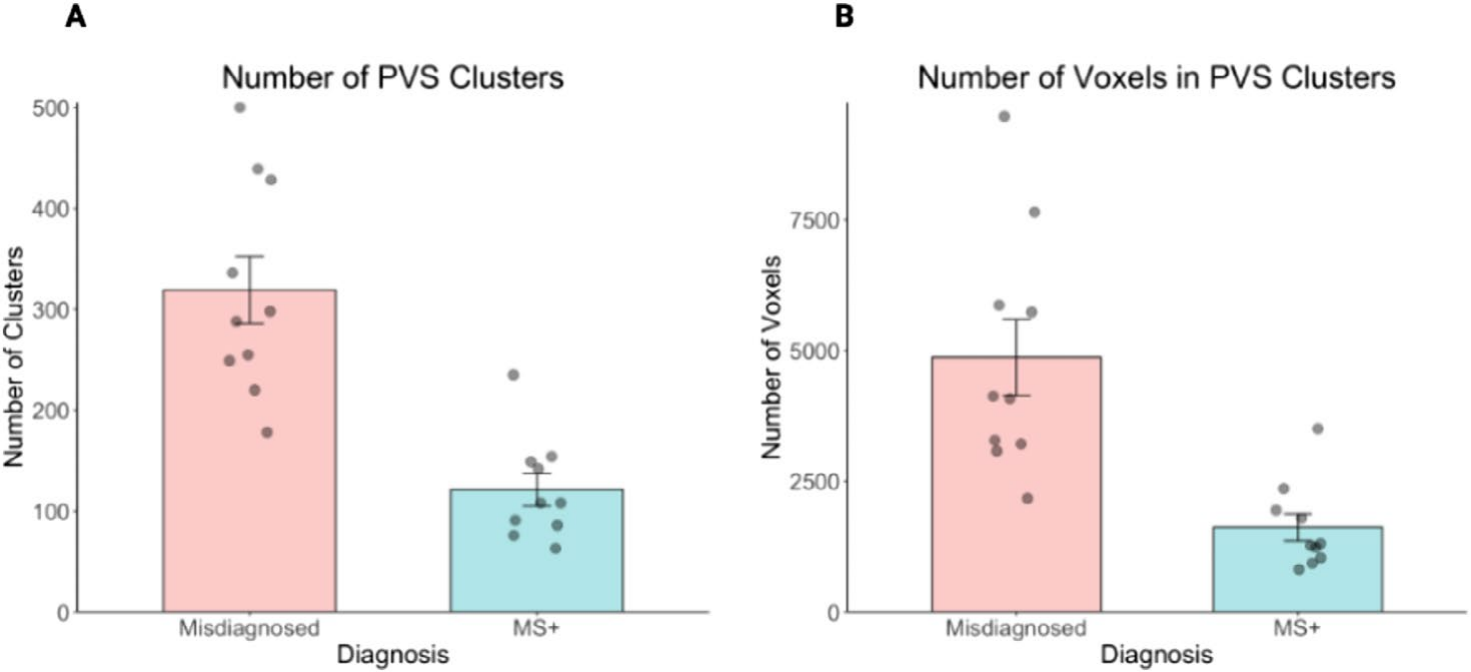
Number of PVS clusters (A) and PVS volume (B) in the misdiagnosed compared to MS+ subgroups.

In subgroup analyses, PVS showed no diagnostic accuracy in distinguishing between MS and the other subgroups. For MS versus MS+, the number of PVS clusters AUC was 0.45, CI [0.18, 0.72]; PVS volume AUC 0.46, CI [0.19, 0.73]. For MS versus migraine, the AUC was 0.64, CI [0.38, 0.90] and 0.71, CI [0.47, 0.95] for number and volume of PVS, respectively. Comparing MS+ and misdiagnosed, the number of PVS and volume showed high diagnostic accuracy, with AUC 0.98, CI [0.93, 1.00] and AUC 0.95, CI [0.86, 1.00] for number and volume of PVS, respectively. Two‐sample *t*‐tests found the MS+ group had significantly fewer PVS clusters and smaller volume than people who were misdiagnosed with MS (*p* < 0.001, point estimate MS+ 121, point estimate misdiagnosed 319, CI [118–277], and *p* < 0.001, point estimate MS+ 1,62, point estimate misdiagnosed 4.87, CI [1.55–4.94]; Figure 3). In logistic regression analysis, the number of PVS and volume was significantly related to the differentiation of MS+ compared to the misdiagnosed group (*p* = 0.029, point estimate −0.04; CI [−0.09, −0.02], and *p* = 0.024, point estimate −2.2, CI [−4.9, −0.80] respectively), with decreasing odds of MS+ diagnosis with increasing PVS number and size. However, this finding did not persist after controlling for age. There were no other significant group differentiations found.

## 4 Discussion

In this study, we found that MS diagnosis was associated with a lower burden of PVS compared to non‐MS diagnoses, and PVS number and total volume had high accuracy for differentiating these groups. Additionally, the odds of having an MS diagnosis decreased as the number and size of PVS increased, a finding that persisted after controlling for age. Although limited by sample size, the subgroup analyses found MS+ had fewer and smaller PVS clusters than the misdiagnosed cohort, and as the number and size of clusters increased, the odds of an MS+ diagnosis decreased compared to the misdiagnosed cohort. Importantly, and unlike prior studies, the non‐MS cohort in this study was not a true control but rather a group with comorbidities known to cause white matter abnormalities on MRI that may mimic MS.

The pathophysiology behind enlarged PVS is not well understood, with proposed causal mechanisms including arterial stiffening, protein aggregation, brain atrophy, and destruction of the BBB [26]. Vascular risk factors may drive these underlying pathological changes through arterial stiffening [27] and protein deposition within vessel walls [28], mechanisms that are well established in small vessel disease [13]. MS may contribute as well, possibly through neuronal loss and interruption of the BBB [29]. At this time, it is not well delineated if enlargement of PVS is driven by vascular disease, neuroinflammation, or perhaps has contributions from both underlying pathologies [29]. This uncertainty is highlighted by existing data surrounding PVS findings in MS, which are conflicting, with some studies showing that PVS in MS are larger and more frequent than in healthy controls [10, 12], while others suggest that enlarged PVS in patients with MS are more likely to be related to underlying microangiopathic change [13, 15]. Our data are supportive of the latter finding, with MS diagnosis being associated with fewer and smaller PVS compared to the non‐MS cohort with risk factors known to be associated with microangiopathic changes.

There were several limitations of this study. The sample size was small, and for the subgroup comparisons, groups were even smaller, limiting the power of this sub‐analysis. There was an absence of a relationship with age and PVS in our cohort, which may be reflective of the relatively narrow age range of participants and small sample size limiting statistical power to detect an association that has been previously described [13]. The lack of difference in PVS burden between MS and MS+ subgroups, and between MS and migraine, may also be best explained by the small sample size of subgroup analysis limiting lower to detect meaningful differences. Grouping non‐MS conditions together may obscure meaningful differences due to heterogeneity in underlying pathologies, and therefore results must be interpreted cautiously in light of the clinical diversity of non‐MS diagnoses. Additionally, in some participants, the possibility of subclinical or undiagnosed comorbidities cannot be excluded. Sex‐related differences were not investigated as there were only three men in the study. As this method for PVS detection has previously been validated with manual ratings [25], we did not perform manual PVS detection. The cross‐sectional study design may also have influenced our findings, as PVS may change throughout the disease course in MS and may be influenced by certain DMTs or their efficacy in control of neuroinflammation. Normal appearing white matter volume was not adjusted for, and although there is a lack of data regarding the relationship between normal appearing white matter volume and PVS burden, decreasing white matter volume as the explanation for there being fewer PVS in MS in this study does not explain why other studies have found more PVS in MS compared to healthy controls. This requires more study to further elucidate, ideally in larger studies with a more homogenous comparator group.

In a cohort of MS patients compared to non‐MS patients with microangiopathic risk factors, we found that MS diagnosis was associated with fewer and smaller PVS. This supports previous findings suggesting that PVS enlargement in MS is more likely to be driven by underlying microangiopathy. Larger future studies in prospective cohorts will be needed to better understand the causes of PVS enlargement in MS.

## Author Contributions

Elle M. Levit participated in conception and design of the study, data analysis and editing. Elizabeth A. Horwath participated in conception and design of the study, data analysis, and editing. Daniel L. Schwartz participated in conception and design of study, editing. Lisa C. Silbert participated in conception and design of study, editing. Russell T. Shinohara participated in conception and design of the study, data analysis and editing. Andrew J. Solomon participated in conception and design of the study, data analysis and editing.

## Ethics Statement

This study was approved by the UVM Institutional Review Board (IRB).

## Consent

Written informed consent was obtained from all participants.

## Conflicts of Interest

Elle M. Levit, Elizabeth A. Horwath, Daniel L. Schwartz, and Lisa C. Silbert declare no conflicts of interest. Russell T. Shinohara reports consulting income from Octave Bioscience and compensation for scientific reviewing from the American Medical Association. Andrew J. Solomon MD reports consulting for Kiniksa Pharmaceuticals and TG Therapeutics. Served on the advisory board for TG Therapeutics, Horizon Therapeutics, Genentech/Roche, Sanofi, and Bristol Meyers Squibb. Provided non‐promotional speaking for EMD Serono. Site PI for contract research for Sanofi, Actelion, Genentech/Roche, Novartis. Received research funding from Bristol Meyers Squibb.

## Data Availability

The data that support the findings of this study are available on request from the corresponding author. The data are not publicly available due to privacy or ethical restrictions.
